# Bacterial regulation of macrophage bacterial recognition receptors in COPD are differentially modified by budesonide and fluticasone propionate

**DOI:** 10.1371/journal.pone.0207675

**Published:** 2019-01-24

**Authors:** Karin A. Provost, Miyuki Smith, Anna Miller-Larsson, Gregory D. Gudleski, Sanjay Sethi

**Affiliations:** 1 Veterans Health Administration, Veterans Affairs Western New York Healthcare System at Buffalo, Division of Pulmonary, Critical Care and Sleep Medicine, Buffalo, New York, United States of America; 2 University at Buffalo, State University of New York, Jacobs School of Medicine and Biomedical Sciences, Division of Pulmonary, Critical Care and Sleep Medicine, Buffalo, New York, United States of America; 3 Respiratory GMed, AstraZeneca Gothenburg, Mölndal, Sweden; 4 University at Buffalo, State University of New York, Jacobs School of Medicine and Biomedical Sciences, Department of Medicine, Buffalo, New York, United States of America; University of Torino, ITALY

## Abstract

**Rationale:**

Patients with COPD have an increased risk for community-acquired pneumonia, which is further increased by inhaled corticosteroids.

**Objective:**

To assess effects of the corticosteroids, budesonide and fluticasone propionate, on macrophage bacterial responses in COPD.

**Methods:**

Monocyte-derived macrophages (MDMs) generated from blood monocytes from 10 non-smoker controls (NoS), 20 smokers without COPD (Sm), and 40 subjects with moderate to severe COPD (21 ex-smokers (COPD-ES) and 19 current smokers (COPD-S)) were pre-treated with budesonide or fluticasone (10 nM—1 μM) and challenged with live non-typeable *Haemophilus influenzae* (NTHI) or *Streptococcus pneumoniae* (SP). Cell surface bacterial recognition receptor expression (flow cytometry) and cytokine release (bead array) were analyzed.

**Results:**

NTHI and SP reduced bacterial recognition receptor expression on MDMs from COPD and Sm, but not NoS (except TLR4). SR-AI and MARCO were reduced by both NTHI and SP, whereas other receptors by either NTHI or SP. Among COPD subjects, COPD-ES demonstrated a greater number of reductions as compared to COPD-S. NTHI reduced SR-AI, MARCO, CD11b, CD35 and CD206 in COPD-ES while only SR-AI and CD11b in COPD-S. SP reduced SRA-1, CD1d, TLR2 and TLR4 in both COPD-ES and COPD-S, and reduced MARCO and CD93 only in COPD-ES. All receptors reduced in COPD by NTHI and most by SP, were also reduced in Sm. Budesonide counteracted the receptor reductions induced by both NTHI (CD206 p = 0.03, MARCO p = 0.08) and SP (SR-AI p = 0.02) in COPD-ES. Fluticasone counteracted only SP-induced reductions in TLR2 (p = 0.008 COPD-ES and p = 0.04 COPD-S) and TLR4 (p = 0.02 COPD-ES). Cytokine release was equivalently reduced by both corticosteroids.

**Conclusions:**

Reduction in macrophage bacterial recognition receptors during bacterial exposure could provide a mechanism for the increased pneumonia risk in COPD. Differential effects of budesonide and fluticasone propionate on macrophage bacterial recognition receptor expression may contribute to the higher pneumonia incidence reported with fluticasone propionate.

## Introduction

Patients with chronic obstructive pulmonary disease (COPD) have an increased risk of community-acquired pneumonia (CAP) that may be further amplified by active smoking[1, 2]. Smokers without COPD also have an increased pneumonia risk[2, 3]. Risks of CAP in COPD are further increased with the use of inhaled corticosteroids (ICS) [4–10], with significantly greater risk of pneumonia observed for fluticasone propionate as compared to other ICS[4, 11–13]. Though ICS reduce exacerbations and improve quality of life in COPD, they do not prevent disease progression[8, 14, 15], and the increased incidence of pneumonia makes their role in COPD treatment increasingly controversial.

Withdrawal of ICS does not entirely reduce pneumonia risk in COPD, as it remains elevated over the normal population[16]. Withdrawal is also associated with reduction in lung function and increased exacerbation frequency[16–19]. The infectious complications associated with ICS use, and the risks associated with ICS withdrawal underscore the need to optimize patient and ICS selection to maximize the risk:benefit ratio.

Mechanistically, glucocorticoids (both oral and inhaled) have immunomodulatory effects through suppression of inflammatory gene transcription and cytokine release from alveolar macrophages and airway epithelial cells[20], that may negatively affect anti-bacterial responses. Inhibitory effects of ICS on bacterial recognition receptor expression, specifically the toll-like receptor family (TLR)[21–23] have been observed in alveolar macrophages and airway epithelial cells, but direct effects on the macrophage response to bacteria have not been investigated.

Our study was designed to explore how CAP-associated bacteria interact with monocyte-derived macrophages (MDMs) from COPD subjects in the presence of ICS. Based on previous studies of impaired macrophage bacterial responses in COPD[24–30], we hypothesized that the macrophage-bacteria interaction in COPD subjects is further impaired if ICS are present, and that the extent of the impairment may differ between ICS products. Utilizing a suspension-cultured blood monocyte-derived macrophage model[30, 31] we studied the effects of budesonide (BUD) and fluticasone propionate (FP) on macrophage responses to non-typeable *Haemophilus influenzae (*NTHI) and *Streptococcus pneumoniae* (SP).

## Methods

### Subject recruitment

The study (AstraZeneca D589BN00039, VAWNY 466402) received Institutional Review Board (IRB) approval from the Veterans Affairs (VA) Western New York Healthcare System at Buffalo. IRB approved, written informed consent was obtained from each subject prior to study entry. Subjects included moderate to severe stable COPD patients (n = 21 ex-smokers (COPD-ES) and n = 19 current smokers (COPD-S)), smokers without COPD (Sm) (n = 20) and non-smoker healthy controls (NoS) (n = 10). Inclusion and exclusion criteria are listed in the supporting information.

### Generation of monocyte-derived macrophages

Monocytes were purified from whole blood by density centrifugation and negative bead depletion[30], and matured in suspension-culture[30, 31] to generate monocyte-derived macrophages. Protocol details provided in the supporting information.

### Monocyte-derived macrophage incubation with bacteria

MDMs were incubated overnight in serum-free HBSS at 37°C, then pre-treated for 24 hours with BUD (AstraZeneca), FP (Sigma) (both in DMSO) or DMSO alone (diluent control) from 10 nM to 1 μM. Live clinical isolates of NTHI(11P6H) or SP(67PP3) were added to the MDMs at log phase (MOI of 200:1 bacteria: MDM) on a rotational shaker at 37°C for 24 hours. BUD, FP or diluent control was continually present. Optimal MOI was determined for cell survival and responses, and consistent with previously published ratios[24, 30]. Cell surface receptor expression and supernatant cytokines were analyzed after 24-hour incubation with bacteria. Details are provided in the supporting information.

### Cytokine analysis

Supernatants from the MDM: bacteria incubation were analyzed by multiplex bead array (BD Biosciences) to determine the concentration of IL-1β, IL-4, IL-6, IL-8, IL-10, IL-12p40, IL-17A, IL-17F, IFN-γ and TNF-α. IL-18 was analyzed by ELISA (eBioscience).

### Flow cytometric analysis of cell surface receptors

Multi-color flow cytometry was used to analyze presence and density of cell surface receptors involved in bacterial recognition including CD1d, CD11b, CD14, CD16, CD35, CD93, CD206, TLR2, TLR4, MARCO and SR-AI as described in the supporting information and S1 Fig.

### Statistical analysis

Statistical analysis was completed using Prism7 (GraphPad) using log-transformed data (due to population skewness) thereby creating a normally-distributed population. All subsequent analyses were therefore parametric, as outlined in the supporting information. Statistically significant differences were defined as p≤ 0.05, after appropriate adjustment for multiple comparisons. Where multiple testing was required, Holm-Sidak (ANOVA) or Benjamini-Hochberg false discovery rate correction (T-test) was done at the specified threshold. Cohen’s-d effect size determination was done to quantify the magnitude of the statistically significant results, given the small sample size. Differences in response to ICS between groups were determined at 10 nM of BUD or FP, as the most clinically relevant and sustained airway and lung tissue concentration after inhalation, and then evaluated for a concentration-response effect from 10 nM to 1 μM. Details provided in the supporting information.

## Results

### Subjects

Aside from expected differences in lung function, NoS and Sm were statistically younger than subjects with COPD. Current smokers of similar advanced age to the COPD subjects frequently had undiagnosed COPD identified at study screening, and entered the study as COPD-S, instead of Sm controls. The difficulty in identifying smokers of similar age who have not already developed COPD is consistent with data that longer duration of tobacco smoke exposure is associated with development of COPD [32, 33] and reflected in similar age differences between groups in similar studies [24, 29, 34, 35]. There was limited availability of NoS controls of similar age to subjects with COPD, who did not meet exclusion criteria.

COPD-S, but not COPD-ES, demonstrated statistically significant higher pack-year tobacco exposure than Sm. There were no statistically significant differences between COPD-ES and COPD-S for degree of airflow limitation, pack-year tobacco exposure or inhaled medication use (Table 1). There were no statistically significant differences in demographics between the control groups of Sm)and NoS (excepting tobacco exposure).

**Table 1:** Subject demographics.

	COPD Ex-Smoker	COPD Smoker	Smoker	Non-smoker	ANOVA	Between Group Comparisons
Number of Subjects (M:F)	21 (20:1)	19 (15:4)	20 (15:5)	10 (5:5)		
Race C:AA:Other	20:1:0	14:5:0	15:5:0	8:1:1		
Age (years) Mean (SD)	69 (9)	63 (9)	50 (11)	44 (13)	p<0.0001	p<0.0001 COPD-ES:Sm p<0.0001 COPD-ES:NoS p<0.0001 COPD-S:NoS p = 0.0005 COPD-S:Sm p = NS COPD-ES:COPD-S p = NS Sm:NoS
FEV1 (%) Mean (SD)	52 (22)	53 (25)	92 (15)	103 (19)	p<0.0001	p<0.0001 COPD-ES:Sm p<0.0001 COPD-ES:NoS p<0.0001 COPD-S:Sm p<0.0001 COPD-S:NoS p = NS COPD-ES:COPD-S p = NS Sm:NoS
FEV1 (L) Mean (SD)	1.7 (0.7)	1.7 (1.0)	3.1 (0.7)	3.4 (1.1)	p<0.0001	p<0.0001 COPD-ES:Sm p<0.0001 COPD-ES:NoS p<0.0001 COPD-S:Sm p<0.0001 COPD-S:NoS p = NS COPD-ES:COPD-S p = NS Sm:NoS
FEV1/FVC (%) Mean (SD)	50 (14)	52 (13)	78 (5)	81 (4)	p<0.0001	p<0.0001 COPD-ES:Sm p<0.0001 COPD-ES:NoS p<0.0001 COPD-S:Sm p<0.0001 COPD-S:NoS p = NS COPD-ES:COPD-S p = NS Sm:NoS
FVC (%) Mean (SD)	76 (21)	78 (22)	88 (23)	102 (20)	p = 0.009	p = 0.01 COPD-ES:NoS p = 0.03 COPD-S:NoS p = NS all other comparisons
FVC (L) Mean (SD)	3.3 (1.2)	3.4 (1.3)	4.0 (0.9)	4.2 (1.6)	p = NS	p = NS all comparisons
Pack year Mean (SD)	62 (45)	71 (50)	37 (24)	——	p = 0.03	p = NS COPD-ES:Sm p = 0.03 COPD-S:Sm p = NS COPD-ES:COPD-S
Baseline medications ICS LABA SABA LAMA SAMA	52% 52% 86% 57% 28%	42% 57% 57% 26% 10%				p = NS for all medications

M: F = Male: Female, FEV1 = Forced expiratory volume in 1 second, COPD-ES = COPD ex-smoker, COPD-S = COPD current smoker, Sm = smoker without COPD, NoS = non-smoker healthy control, C = Caucasian, AA = African American, ICS = inhaled corticosteroid, LABA = long-acting β_2_-adrenergic agonist, SABA = short-acting β_2_-adrenergic agonist, LAMA = long-acting muscarinic antagonist, SAMA = short-acting muscarinic antagonist.

Analysis by one-way ANOVA for baseline demographics between experimental groups (COPD-ES: COPD-S: Sm: NoS) with Holm-Sidak correction for multiple comparisons. Unpaired T-test for comparison of COPD-ES to COPD-S for medication use. There were no differences between COPD-ES and COPD-S for any demographic parameter.

### Baseline bacterial recognition receptor expression was increased on MDMs from COPD subjects and healthy smokers as compared to non-smokers

**Fig 1:**
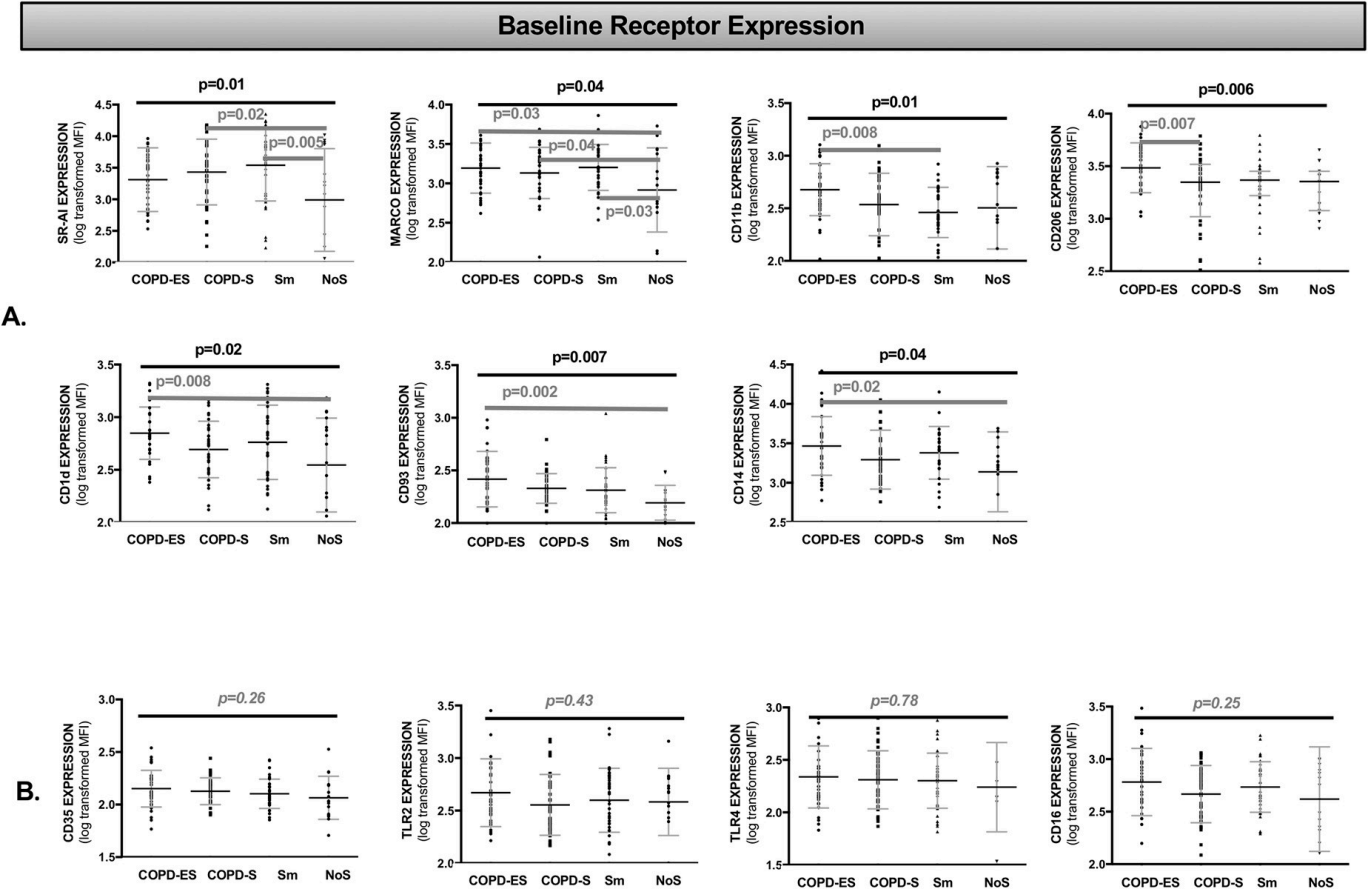
Cell surface bacterial recognition receptor expression at baseline was greater on MDMs from subjects with COPD and healthy smokers as compared to non-smokers. Analysis by one-way ANOVA for receptor MFI (mean fluorescence intensity), representative of cell surface receptor density, presented as an aligned dot-plot with thin black bar at the mean, flanking short grey bars delineating the SD. Thick black horizontal bars above the entire graph mark the ANOVA results of statistical significance. Thick dark grey horizontal bars start and end at the comparisons of statistical significance identified in multiple comparison analysis (A). The bottom row (B) presents the receptors that did not have significant differences in cell surface expression between groups at baseline. Holm-Sidak correction was performed for multiple comparisons. COPD-ES = COPD ex-smoker, COPD-S = COPD current smoker, Sm = smoker without COPD, NoS = non-smoker healthy control, SR-AI = Scavenger receptor A-I, MARCO = Macrophage receptor with collagenous structure, TLR = Toll like receptor. S2 Fig demonstrates the Fig 1A differences in baseline receptor expression in heat map format.

To determine whether differences in baseline bacterial recognition receptor expression could explain impaired macrophage bacterial responses in COPD[24–30], we evaluated cell surface bacterial recognition receptor expression in MDMs. As compared to NoS, untreated, unchallenged MDMs from COPD-ES subjects demonstrated significantly greater expression of the scavenger receptor MARCO (p = 0.03), CD1d (non-classical MHC, lipid antigen presentation, p = 0.008), CD93 (calcium ion binding, protein and carbohydrate ligand recognition, C1qR complex, p = 0.002) and CD14 (lipoteichoic acid (LTA) and lipopolysaccharide (LPS) ligand recognition, p = 0.02) and approached statistical significance for scavenger receptor SR-AI (p = 0.06) (Figs 1A and S2).

COPD-S demonstrated significantly greater expression of the scavenger receptors SR-AI (p = 0.02) and MARCO (p = 0.04) as compared to NoS. When COPD-S were compared to COPD-ES, COPD-ES had significantly greater baseline expression of mannose receptor CD206 (p = 0.007) than COPD-S (Figs 1A and S2).

Importantly, there were no receptors with lower baseline expression in COPD-ES or COPD-S as compared to NoS controls as might have been expected given previously identified impairments in bacterial phagocytosis[24, 34]. The expression of four receptors, CD35 (complement receptor CR1), TLR2, TLR4 (pattern recognition receptors) and CD16 (low affinity receptor for IgG), were not significantly different in COPD-ES or COPD-S (Fig 1B) as compared to NoS.

The effect of active tobacco smoke exposure on baseline receptor expression was investigated comparing COPD-S and Sm with NoS. SR-AI and MARCO demonstrated significantly increased baseline expression in both COPD-S and Sm compared to NoS (Figs 1A and S2). There were no statistically significant differences in baseline receptor expression between COPD-S and Sm.

### Bacterial challenge with NTHI and SP led to reductions in bacterial recognition receptor expression on MDMs from COPD subjects and healthy smokers

To determine effects of bacterial exposure on COPD macrophage responses, MDMs were challenged with NTHI or SP and post-challenge bacterial recognition receptor expression was compared to unstimulated control MDMs (baseline). After bacterial exposure, COPD MDMs had significantly reduced expression of bacterial recognition receptors, that was not consistently observed in NoS. Only TLR4 was reduced in NoS, by NTHI (p = 0.01) and SP (p = 0.001).

**Fig 2:**
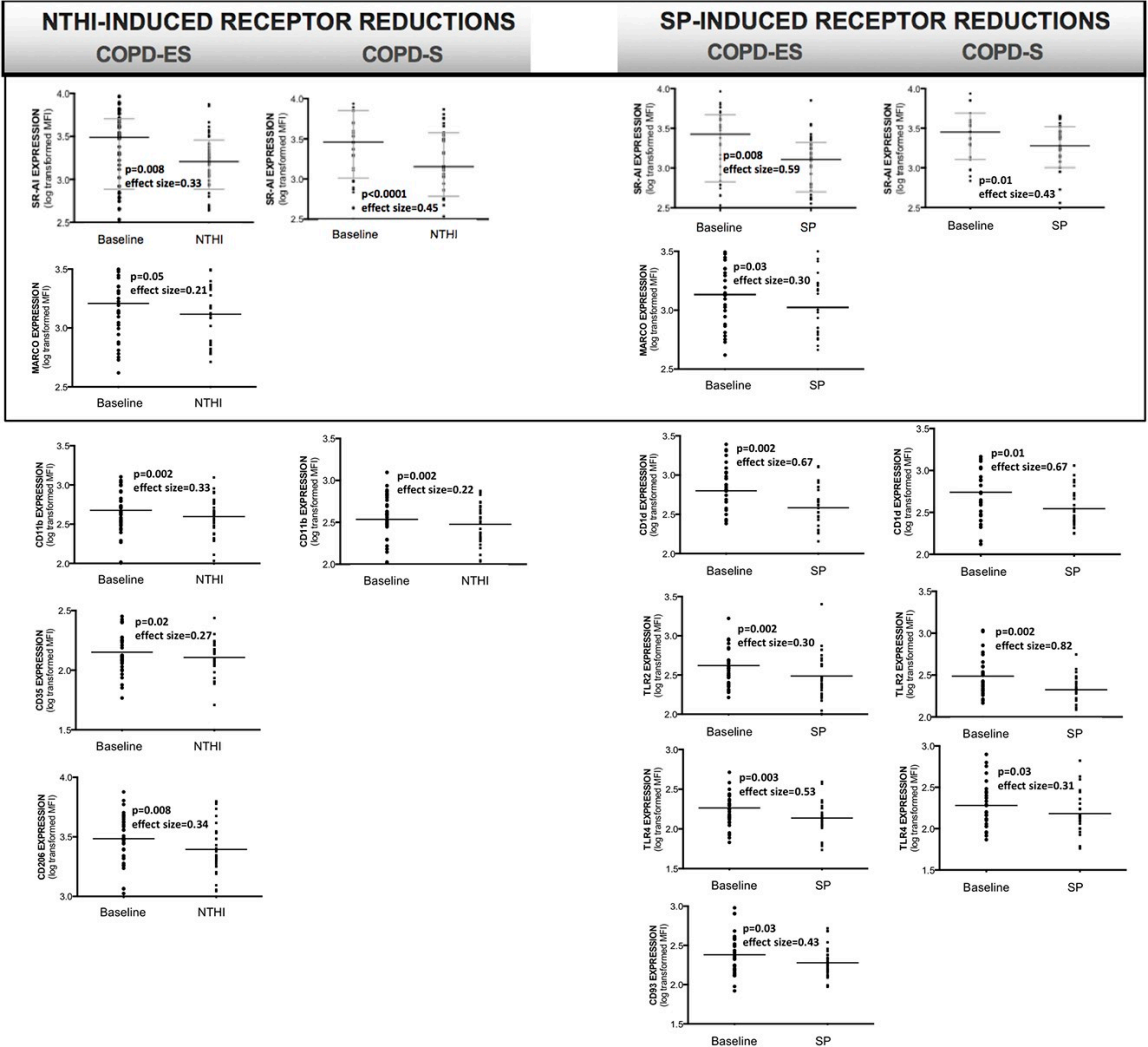
NTHI and SP-mediated reductions of bacterial recognition receptor expression on MDMs from subjects with COPD. Analysis by paired T-test of log-transformed receptor MFI (mean fluorescence intensity) representative of cell surface receptor density, as compared to baseline receptor expression. Boxes around SR-AI and MARCO denote the only receptors reduced by both NTHI and SP. Receptor expression is presented as an aligned dot-plot with thin black bar at the mean, flanking short grey bars delineating the SD. Effect size calculated by Cohen’s-d calculation. S3 Fig provides additional heat map format of statistically significant NTHI- and SP-induced reductions in bacterial recognition receptors.

In subjects with COPD, COPD-ES had a greater number of receptors reduced by bacterial exposure as compared to COPD-S (Fig 2, Table 2). SR-AI expression was most impacted, with statistically significant reductions induced by both NTHI and SP, in COPD-ES and COPD-S. Both NTHI and SP also reduced MARCO, though only in COPD-ES. Beyond their common negative effects on scavenger receptor SR-AI and MARCO expression, both NTHI and SP each mediated additional and distinct reductions in bacterial recognition receptor expression in subjects with COPD; NTHI significantly reduced expression of complement and adhesion receptor CD11b in both COPD-ES and COPD-S, and complement receptor CD35, mannose pattern recognition receptor CD206 only in COPD-ES. SP significantly decreased expression of CD1d, pattern recognition toll-like receptors TLR2 and TLR4 in both COPD-ES and COPD-S, and CD93 in COPD-ES only (Figs 2 and S3, Table 2).

**Table 2:** NTHI and SP-mediated reductions in bacterial recognition receptor expression on MDMs from subjects with COPD and smokers.

**Receptor**	COPD Ex-Smoker	COPD Smoker	Healthy Smoker	COPD Ex-Smoker	COPD Smoker	Healthy Smoker
**SRA-I**	**p = 0.008**	**p<0.0001**	**p<0.0001**	**p = 0.008**	**p = 0.01**	**p<0.0001**
**MARCO**	**p = 0.05**	*p = 0*.*61*	**p = 0.007**	**p = 0.03**	*p = 0*.*44*	*p = 0*.*07*
**CD11b**	**p = 0.002**	**p = 0.002**	**p = 0.004**	*p = 0*.*44*	*p = 0*.*34*	*p = 0*.*99*
**CD35**	**p = 0.02**	*p = 0*.*25*	**p<0.0001**	*p = 0*.*13*	*p = 0*.*16*	**p = 0.04**
**CD206**	**p = 0.008**	*p = 0*.*21*	**p = 0.0001**	*p = 0*.*25*	*p = 0*.*73*	*p = 0*.*96*
**CD1d**	*p = 0*.*06*	*p = 0*.*31*	**p = 0.004**	**p = 0.002**	**p = 0.01**	**p<0.0001**
**TLR2**	*p = 0*.*52*	*p = 0*.*56*	**p = 0.03**	**p = 0.002**	**p = 0.002**	*p = 0*.*06*
**TLR4**	*p = 0*.*13*	*p = 0*.*83*	**p = 0.001**	**p = 0.003**	**p = 0.03**	**p = 0.001**
**CD93**	*p = 0*.*10*	*p = 0*.*08*	**p = 0.002**	**p = 0.03**	*p = 0*.*72*	*p = 0*.*25*
**CD14**	*p = 0*.*11*	*p = 0*.*08*	**p<0.0001**	*p = 0*.*85*	*p = 0*.*52*	*p = 0*.*58*
**CD16**	*p = 0*.*19*	*p = 0*.*73*	*p = 0*.*29*	*p = 0*.*11*	*p = 0*.*09*	*p = 0*.*10*

Analysis by paired T-test of log-transformed receptor MFI (mean fluorescence intensity) representative of cell surface receptor density after NTHI or SP exposure, as compared to baseline receptor expression with representative p-values listed. Light grey shading for SR-AI and MARCO denote receptors reduced by both NTHI and SP. Italicized p-values did not reach statistical significance. Benjamini-Hochberg correction for multiple comparisons (false discovery rate) was done, accepting a threshold of 0.10 given the exploratory nature of the study, which was consistent with uncorrected p-values meeting the pre-specified significance level of ≤0.05.

When we evaluated the effect of tobacco smoke on changes in receptor expression induced by NTHI or SP on COPD-S and Sm MDMs, Sm had a greater number of receptors that were significantly reduced by NTHI (all measured receptors except CD16) as compared to COPD-S (SR-AI and CD11b only) (Table 2). SP-mediated statistically significant reductions in both Sm and COPD-S were observed for SR-AI, CD1d, and TLR4, and additional significant reductions were observed for CD35 in Sm, and TLR2 in COPD-S.

Given the statistically significant differences in mean age between the experimental groups (COPD groups were older than Sm and NoS), linear regression analyses of changes in receptor expression as a function of age were performed and adjusted for multiple comparisons using the Benjamini-Hochberg false discovery rate (FDR) correction (0.05 threshold). There was no statistically significant effect of age on NTHI- or SP-induced changes in MDM expression of bacterial recognition receptors. Given the number of receptors evaluated by independent paired t-test (response to bacteria exposure), Benjamini-Hochberg false discovery rate (FDR) correction (threshold of 0.10, given the exploratory nature of the study) was performed, and did not change which receptor responses reached statistical significance.

### Budesonide prevented reductions in bacterial recognition receptors by NTHI and SP

To determine the impact of ICS treatment on COPD MDM responses to NTHI and SP, we compared receptor expression on MDMs pre-treated with BUD or FP to diluent-control treated MDMs. ICS effects on receptor expression were analyzed at ICS concentration of 10 nM (reflecting concentrations sustained in the lung after inhalation) and then assessed for a concentration-response relationship (linear trend) at 10 nM, 100 nM and 1 μM concentrations. MDM responses to NTHI and SP were significantly different depending on the pre-treatment ICS.

**Fig 3:**
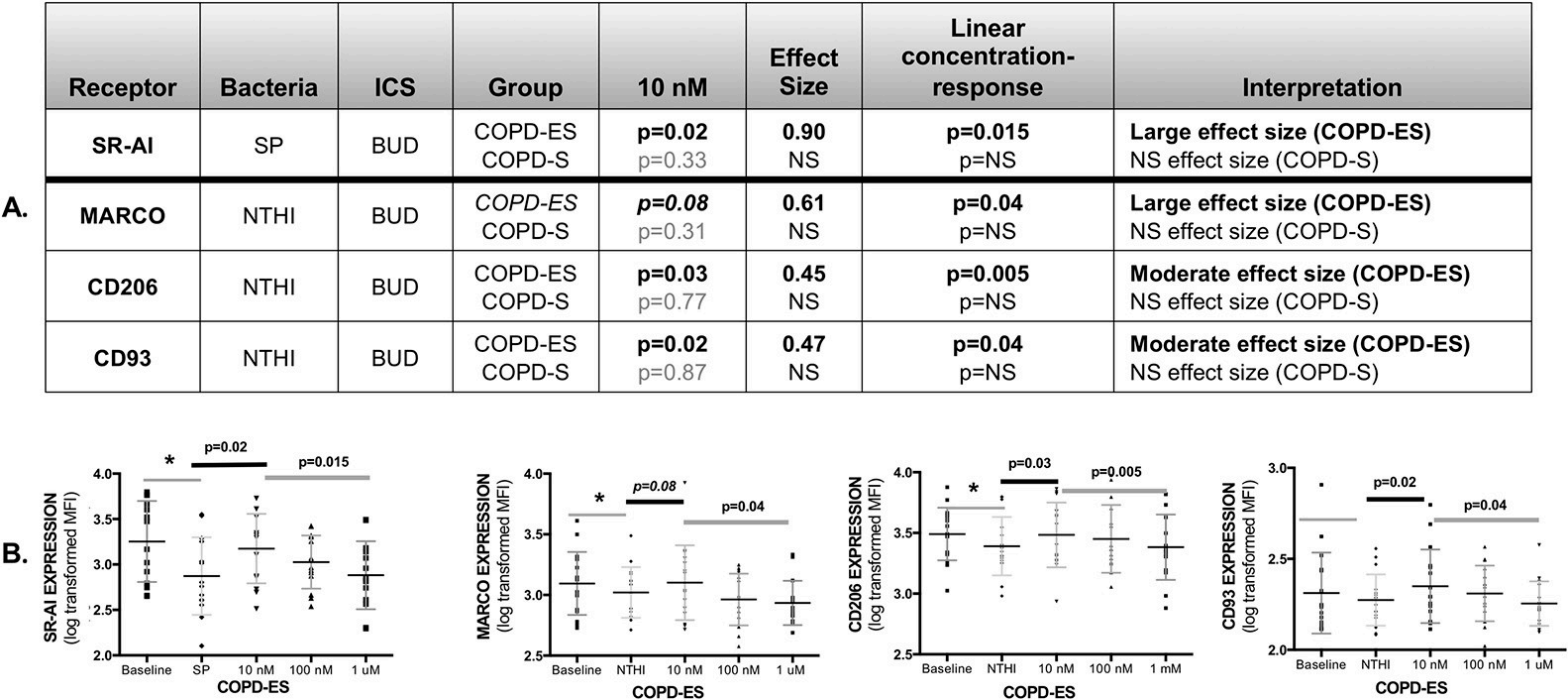
Budesonide increased bacterial recognition receptor expression on MDMs from COPD ex-smokers for both NTHI and SP with greatest receptor upregulation at 10 nM, reflecting an inverse concentration-response relationship. Analysis of log-transformed data on receptor MFI (mean fluorescence intensity). The p-values were calculated by paired T-test, for effect of budesonide (BUD) at 10 nM on SP- or NTHI mediated reduction of receptor expression on MDMs from COPD ex-smokers (COPD-ES) and COPD current smokers (COPD-S). Effect size for BUD effect on MDM receptor expression at 10 nM was calculated by Cohen’s-d calculation. Italicized p-value approached statistical significance, and is reported due to the large effect size. Light grey p-values indicate a statistically not-significant result (NS), and subsequent effect size and concentration-response values are shown as NS **(A, Table).** Receptor expression is presented as an aligned dot-plot with thin black bar at the mean, flanking short grey bars delineating the SD **(B, Figures)**. Significant p-values for the BUD effect on receptor expression at 10 nM are noted above the thick, short black bar. Linear trend analysis (denoted by thick dark grey line) was done for concentration-response relationship between 10 nM, 100 nM and 1 μM of BUD calculated by repeated measures one-way ANOVA. Significant reductions in baseline receptor expression by NTHI or SP are noted by an asterisk (*), representing significant data presented in manuscript Fig 2, and S3 Fig. BUD increased expression of CD93 in NTHI-exposed MDMs although CD93 expression was not reduced by NTHI. NS = not statistically significant.

BUD pre-treatment of MDMs from COPD-ES prevented reductions in bacterial recognition receptors by both NTHI and SP, with an inverse concentration-response relationship, showing greatest effect at 10 nM. BUD significantly prevented reductions in SR-AI by SP, and NTHI-mediated reductions in CD206, and approached statistical significance for NTHI-induced reduction in MARCO (Fig 3). BUD increased expression of CD93 during NTHI challenge, though NTHI did not specifically decrease expression of this receptor. The improvements in receptor expression by BUD were restricted to COPD-ES; BUD did not improve receptor expression on MDMs from COPD-S.

### Fluticasone prevented reductions in bacterial recognition receptors only by SP

**Fig 4:**
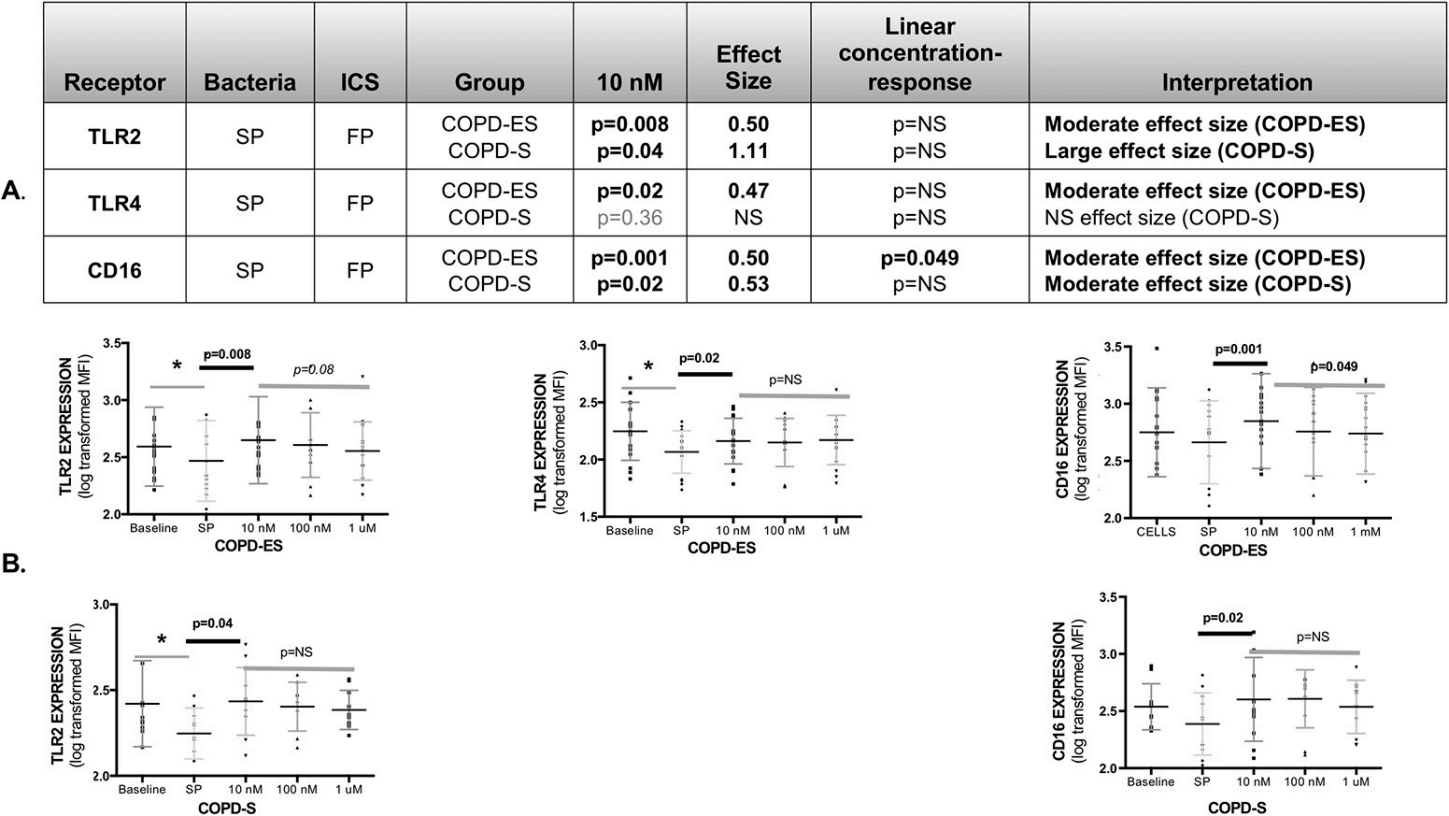
Fluticasone propionate increased bacterial recognition receptor expression on MDMs from subjects with COPD for SP only, with greatest receptor upregulation at 10 nM, reflecting an inverse concentration-response relationship. Analysis of log-transformed data on receptor MFI (mean fluorescence intensity). The p-values were calculated by paired T-test, assessing the effect of fluticasone propionate (FP) at 10 nM on SP-induced reduction of receptor expression on MDMs from COPD ex-smokers (COPD-ES) and COPD current smokers (COPD-S). Effect size for FP effect on MDM receptor expression at 10 nM was calculated by Cohen’s-d calculation **(A, Table)**. Only CD16 demonstrated a significant concentration–response relationship (inverse) to FP. Receptor expression is presented as an aligned dot-plot with thin black bar at the mean, flanking short grey bars delineating the SD **(B, Figures)**. Significant p-values for fluticasone effect on receptor expression at 10 nM are noted above the thick short black bars. Linear trend analysis (denoted by thick dark grey line) was done for concentration-response relationship between 10 nM, 100 nM and 1 μM of FP calculated by repeated measures one-way ANOVA. Significant reductions in baseline receptor expression by SP are noted by an asterisk (*), representing significant data presented in manuscript Fig 2 and S3 Fig. Of note, FP increased expression of CD16 in SP-exposed MDMs although CD16 expression was not reduced by SP. NS = not statistically significant.

FP at 10 nM also prevented reductions in bacterial recognition receptor expression, but this was limited to SP-mediated reductions in TLR2 expression (COPD-ES and COPD-S), and TLR4 expression (COPD-ES only), with no statistically significant concentration-response relationship (Fig 4). Although CD16 was not significantly reduced by bacteria, FP increased CD16 expression above baseline levels in SP-challenged MDMs from COPD-ES and COPD-S, with an inverse concentration-response relationship only for COPD-ES.

### ICS reduced bacterial recognition receptor expression only on MDMs from COPD-S during NTHI exposure

Only CD11b and CD1d were reduced on MDMs from COPD-S by the presence of ICS during NTHI exposure. CD11b was reduced by NTHI, and further reduced by FP in COPD-S (p = 0.01). Though not initially reduced by NTHI, CD1d was reduced in COPD-S by BUD (p = 0.01).

### NTHI and SP-induced cytokine release from MDMs did not significantly differ between subjects with COPD, Sm and NoS

Cytokine concentrations in supernatants from untreated, unchallenged MDMs were undetectable or detected in very low levels. Cytokine release from MDMs in response to NTHI and SP was not impacted by disease or smoking status, as there were no statistically significant differences in cytokine release from MDMs from COPD-ES, COPD-S, Sm or NoS after stimulation with either NTHI or SP in the absence of ICS. The cytokines IL-4, IL-10, IL-12p40, IL-17A, IL-17F and IFN-γ were not detected in culture supernatants at baseline or after bacterial challenge.

### Both budesonide and fluticasone propionate significantly reduced NTHI-induced release of IL-1β, IL-6, IL-8 and TNF-α from COPD MDMs

**Fig 5:**
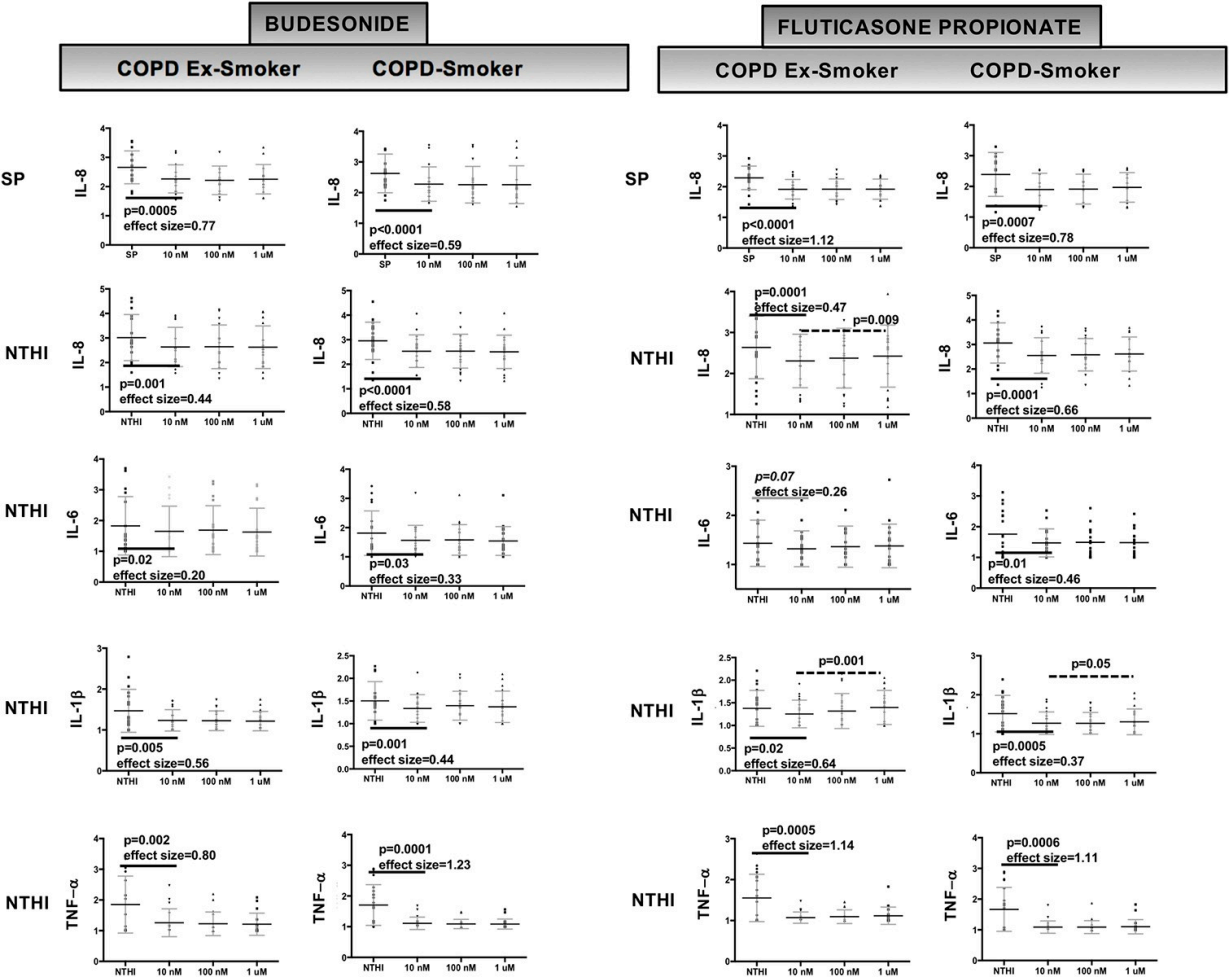
Budesonide and fluticasone propionate both effectively reduced cytokine release from COPD MDMs exposed to NTHI and SP. Analysis of log-transformed data on cytokine concentrations (pg/mL) in MDM-bacteria-ICS co-culture supernatants. The p-values were calculated by paired T-test, assessing the effect of budesonide (BUD) or fluticasone propionate (FP) at 10 nM on NTHI- or SP-induced cytokine release by MDMs from COPD ex-smokers (COPD-ES) and COPD current smokers (COPD-S). Concentration of cytokine released at each concentration of ICS is presented as an aligned dot-plot with thin black bar at the mean, flanking short grey bars delineating the SD. Significant p-values for ICS effect on cytokine release (at 10 nM) and effect size are noted below the thick solid black bars. Only FP inhibition of NTHI-stimulated release of IL-1β from COPD-ES and COPD-S, and NTHI-stimulated release of IL-8 in COPD-ES demonstrated a statistically significant concentration-response relationship (inverse, greatest suppression at 10 nM) and is denoted by a dashed line over the concentrations with p-value above line. Extent of inhibition of cytokine release was not significantly different between BUD and FP and did not reach EC50 value for any cytokine. Effect size calculated at 10 nM by Cohen’s-d calculation.

Release of IL-1β, IL-6, IL-8 and TNF-α in response to NTHI demonstrated statistically significant inhibition in MDMs from COPD-ES and COPD-S by both BUD and FP, although the effect of FP on IL-6 release in MDMs from COPD-ES only approached statistical significance (p = 0.07)(Fig 5). Despite moderate to large effect sizes and statistically significant reductions in cytokine release, none of the reductions in cytokine release reached EC50 for either BUD or FP (Fig 5).

IL-18 release after NTHI challenge was not reduced by either BUD or FP for COPD-ES or COPD-S (p>0.05 for all comparisons).

### Both budesonide and fluticasone propionate significantly reduced only SP-induced IL-8 release from COPD MDMs

SP-induced IL-8 release was statistically significantly reduced by both BUD and FP in MDMs from COPD-ES and COPD-S with large effect sizes, but did not reach EC50 (Fig 5). SP-induced release of IL-6 and IL-18 was not reduced by either BUD or FP for COPD-ES or COPD-S (p>0.05 for all comparisons).

### Concentration-response relationships were not consistently observed for cytokine reduction by budesonide or fluticasone propionate

A statistically significant linear concentration-response effect (inverse) was observed only for FP inhibition of NTHI-stimulated release of IL-1β in MDMs from COPD-ES and COPD-S, and NTHI-stimulated release of IL-8 in MDMs from COPD-ES, with the greatest suppression at 10 nM)(Fig 5). BUD and FP demonstrated approximately equal cytokine reduction at all concentrations (10 nM to 1 μM) for all other cytokines (Fig 5).

## Discussion

To the best of our knowledge, we are the first to show statistically significant reductions in bacterial recognition receptor expression induced by pathogenic bacteria associated with CAP (NTHI and SP), affecting MDMs from COPD-ES, COPD-S and smokers without COPD (Sm). Healthy non-smoker (NoS) MDMs did not reduce expression of bacterial recognition receptors excepting TLR4, whose expression can be negatively regulated, independent of COPD, by ligand engagement through effects on gene expression[22, 36] and direct receptor cleavage[37].

COPD-ES were more significantly affected in terms of the number of NTHI and SP mediated reductions in receptor expression as compared to COPD-S, despite similar age, cumulative pack year tobacco status and disease severity. Ongoing tobacco smoke exposure alone did not account for the differences between COPD-ES and COPD-S, as Sm had a greater number of receptor reductions as compared to COPD-ES and COPD-S after NTHI, and fewer reductions after exposure to SP. Consistent with the significant number of bacterial recognition receptors reduced in Sm after NTHI exposure, others have also identified deficits in immune responses of smokers to NTHI (impaired phagocytosis) but not SP [29]. The specificity of the reductions observed in MDMs involving COPD-ES, COPD-S and Sm, but not NoS, is suggestive of impairments in macrophage anti-bacterial responses that begin during tobacco exposure, even before the development of formal airflow obstruction (COPD).

The reductions in receptor expression after bacterial challenge likely to have the greatest impact on airway bacterial immunity are those reduced by both NTHI- and SP, reflecting broadly compromised ligand recognition, and affecting Sm, COPD-S and COPD-ES. We hypothesize that the impairment involving both Sm, and all COPD subjects may reflect an early (Sm) and sustained impairment in macrophage function (COPD-S and COPD-ES). Receptors reduced in Sm, COPD-S and COPD-ES but limited to either NTHI (complement receptor CD11b), or SP (lipid antigen receptor CD1d and TLR4) may also reflect early and sustained alterations in immunity specific to individual bacteria or class associated with CAP. Bacterial recognition receptor reduction after exposure of COPD MDMs to NTHI or SP also appeared to impact immune responses specific each bacteria/class. Disease state (Sm, COPD-S, COPD-ES) did appear to impact the receptor expression, with the fewest receptor reductions observed on COPD-S MDMs during bacterial exposure, and is best represented visually in Table 2.

Although further work is needed to dissect the impact and mechanism of these reductions, our identification of reduction in expression of key macrophage bacterial recognition receptors in response to NTHI and SP challenge observed in Sm and COPD subjects may provide a mechanistic explanation for the increased risk of CAP that has been observed in these patients[2, 3].

We also observed increased baseline expression of bacterial recognition receptors in unstimulated MDMs from COPD-ES, COPD-S and Sm as compared to NoS, which may reflect differences in the baseline polarization state associated with disease state. Although matured *ex vivo*, the differences we observed between COPD-ES, COPD-S and Sm as compared to each other and NoS suggests that the *ex vivo* suspension culture maturation process preserves key differences in macrophage function and phenotype between the groups. The pattern of baseline scavenger receptor expression that we observed in subjects with COPD and Sm were consistent with a mixed M1 (increased SR-AI expression) and M2 phenotype (increased MARCO and reduction in SR-AI), with statistically significant increases in expression of both SR-AI and MARCO in Sm and COPD-S as compared to NoS, and only MARCO in COPD-ES (though SR-AI is still elevated in COPD-ES above NoS, this difference did not reach statistical significance). Increased baseline expression of CD206 (M2 associated) and MARCO in COPD-ES, may suggest a shift toward a more M2 phenotype in COPD-ES as compared to COPD-S. The short duration of our model system (24 hours post bacterial exposure) may have captured only the start of the transition.

Our cytokine data also favors a mixed M1/M2 phenotype that was not significantly different between COPD-ES, COPD-S, Sm and NoS, with release of IL-1β, IL-6, IL-8 and TNF-α after bacterial challenge, and undetectable IL-4, IL-10, IL-12 and interferon-γ. The effect of GM-CSF during suspension-culture maturation, now recognized to influence macrophages toward an M1-phenotype, was removed from the culture system for 48 hours prior to baseline receptor determination and bacterial stimulation, minimizing the pro-M1 effect in our model.

Although provocative, our data on the alteration in bacterial recognition receptor expression at baseline and in response to bacterial challenge, cannot be directly extrapolated to the alveolar macrophage. Blood monocytes have been observed to have higher baseline expression of bacterial recognition receptors TLR2, TLR4 and some scavenger receptors (CD36, CD61, CD14)[38, 39] as compared to alveolar macrophages in COPD subjects (both current and former smokers). Further study is required to determine whether baseline elevations of the receptors we have identified as upregulated on MDMs from COPD subjects, are also increased in alveolar macrophages, though complement receptor 3 (CR3) and CR4 expression on AM have been shown to have higher baseline expression in subjects with COPD (both current and ex-smokers) as compared to non-smoker controls[35, 40, 41]. Whether these receptors will downregulate after bacterial challenge in alveolar macrophages, similarly to the MDMs, is also unknown, particularly for the key scavenger receptors SR-AI and MARCO, though reductions have been observed in murine models[42]. The current data on responses of bacterial recognition receptors on human alveolar macrophages to bacterial exposure in COPD is limited to a single study, where TLR2 and TLR4 increased expression after challenge with live *Moraxella catarrhalis* (MC), NTHI and SP in adherent alveolar macrophages[26].

The presence of BUD or FP during bacterial challenge, restored cell surface expression of some of the key bacterial recognition receptors that were reduced by NTHI or SP exposure, which was an unexpected finding. However, we observed significant differences between the effects of BUD and FP, suggesting product-specific, rather than drug class effects. BUD pre-treatment of MDMs from COPD-ES counteracted the bacterial reductions in receptor expression for both SP (SR-AI) and NTHI (CD206), and approached statistical significance for neutralizing NTHI reductions of MARCO. BUD also significantly increased CD93 expression in COPD-ES during NTHI challenge. All receptors increased by BUD demonstrated a significant inverse concentration-response relationship (linear trend) between 10 nM, 100 nM and 1 μM. The greatest effects were observed at 10 nM, which is closest to the lung concentration sustained for several hours after drug delivery (1.0–1.5 log higher concentrations can be reached initially after inhalation)[43]. BUD did not have a significant effect on TLR2 or TLR4 expression in our MDM model, however studies in alveolar macrophages have shown that 10 nM BUD, alone or with TLR2/TLR4 ligands, could upregulate TLR2 gene expression in smokers with/without COPD[22]. The ability of BUD to increase expression of key receptors on both MDMs (SR-AI, MARCO, CD206, CD93) and alveolar macrophages (TLR2), that have a significant role in bacterial recognition (SR-AI, MARCO, CD206)[44–46] and recognition of apoptotic cells (CD93), immune responses that have been identified as impaired in COPD[27], may suggest a role for BUD in improving macrophage function in COPD, beyond anti-inflammatory effects.

The effects of FP on receptor expression were limited to restoration of SP-mediated receptor reductions of TLR2 and TLR4, and increasing CD16 above baseline; though effects were observed in both COPD-ES and COPD-S (TLR2 and CD16). FP had no effect on NTHI-induced receptor reductions. The restoration of TLR2 and TLR4 expression by FP did not demonstrate a concentration-response relationship, however upregulation of CD16 above baseline levels had a similar inverse concentration-response relationship in COPD-ES as was observed with BUD. The effects of FP on receptors associated with pneumococcal recognition (TLR2, and TLR4 ligand of pneumolysin) suggests improved responsiveness to SP. The lack of any upregulatory effect of FP on NTHI-mediated reductions in MDM receptor expression is an important distinction between the two ICS products as NTHI infection has a significant role not only in CAP, but also in acute COPD exacerbations and chronic airway bacterial colonization in advanced COPD.

The inverse concentration-responses observed for the effects of ICS on bacterial recognition receptor expression in the range between 10 nM and 1 μM may represent the descending part of the bell-shaped concentration-response curve, with the top around 10 nM. The bell-shaped curve of glucocorticoid action reflects that low and moderate levels of glucocorticoids (in the range of unstressed plasma cortisol) are optimal to stimulate immune defense mechanisms, whereas higher concentrations are anti-inflammatory (suppressive) and may be protective against exaggerated inflammatory responses[47]. The reason for the lack of concentration-response of FP on TLR2 and TLR4 receptors is unclear, but suggests a plateau for the concentration range.

We recognize that as a class, oral and inhaled corticosteroids have pleiotropic effects on immune and airway epithelial cells in the lung. The ability of BUD and FP to increase cell surface expression of key bacterial recognition receptors was an unexpected finding, and once better understood, may be able to improve macrophage responses to bacterial infection (bacterial recognition, phagocytosis), that may offset the other immunosuppressive effects of ICS. As BUD and FP differed in their ability to augment bacterial recognition receptor expression, the greater effects of BUD in restoring bacterial recognition receptor expression from both NTHI- and SP-induced reductions (though limited to COPD-ES), and the lack of effect of FP on NTHI-mediated receptor effects, may provide a mechanism to explain the increased frequency of pneumonia observed in studies where FP was used, as compared to BUD and other ICS preparations[4, 7, 11–13].

Both BUD and FP significantly and approximately equally reduced cytokine release from MDMs during bacterial challenge with SP and NTHI. The similar reductions in cytokine release by both BUD and FP, that did not achieve EC50 at concentrations sustained in the lung, suggest that the differential risk of pneumonia observed with ICS use in COPD is more likely mediated by the differences on MDM bacterial recognition receptor expression and the downstream effects on bacterial recognition and phagocytosis.

Our study has several limitations, including the fact that we had insufficient cell numbers to investigate whether the presumed protective effects of BUD and FP on several bacterial recognition receptors translated to improved phagocytosis, though others have shown BUD to improve phagocytosis of NTHI and SP in MDMs from COPD subjects(29). Although the suspension-cultured MDMs more consistently replicate many of the key deficits observed in alveolar macrophages[30, 31] as compared to adherence maturation[24, 29], they cannot fully account for the regional lung environment, and our data must be confirmed in invasively sampled AMs. Because there can be variability in results between adherence and suspension-maturation of blood monocytes, this makes direct comparison of MDM studies difficult. Another limitation was that our control groups were younger than the diseased, and age can affect immune responses. However, we were able to find any correlation of age to the immune response parameters that were examined in this study. Other studies in this area of research have had similar challenges in matching age among groups, and have consistently shown a similar lack of impact of age on their outcomes[24, 29, 34]. Our work does advance the understanding of macrophage anti-bacterial responses as we used live, patient-acquired bacterial strains, rather than heat-killed[29], which more effectively replicates acute respiratory infection. Our current and future work, investigating the beneficial and detrimental effects of ICS on lung defense mechanisms, could lead to more appropriate and targeted ICS use in COPD, and spur development of more selective corticosteroids or non-steroidal glucocorticoid receptor agonists.

## Supporting information

S1 Fig Receptor expression in COPD MDMs in response to NTHI or SP and ICS. Histograms represent surface expression receptors gated on live COPD MDMs at baseline (brown histogram), after exposure to bacteria (green histogram) and ICS effects on receptor expression during exposure to bacteria (pink histogram), presented as mean fluorescence intensity (MFI). This is as described in the main text in Fig 2 (BUD) and Fig 3 (FP). X-axis is shown as the MFI, representative of the number of receptors per cell. Y axis represents the number (as percent) of the total number (Max).
The **top row** demonstrates baseline MDM receptor expression (brown histogram) in MDMs not exposed to bacteria or ICS, as compared to unstained and unstimulated MDMs (cellular autofluorescence, blue histogram, biologic comparator). The black horizontal line and the number above reflect the gate used to determine the percent of cells with receptor expression detectable above cellular autofluorescence.
The **second row** shows the effect of bacterial exposure on MDM receptor expression (green histogram) in relation to baseline expression (brown histogram) and autofluorescence (blue histogram) in unstained MDMs. SR-AI, TLR2, TLR4, CD16 represent the effect of SP on receptor expression; MARCO, CD206, CD93 represent effects of NTHI.
The **third row** demonstrates receptor expression on MDMs pretreated with ICS and exposed to bacteria (pink histogram) in relation to receptor expression on MDMs not treated with ICS before bacterial exposure (green histogram). Effect of BUD is shown for SR-AI, MARCO, CD206 and CD93 (first 4 histograms, from left to right). Effect of FP is shown for TLR2, TLR4 and CD16 (last 3 histograms, from left to right).
The tables in the bottom row provide the MFI for (from top to bottom) 1) unstained, unstimulated cells, 2) baseline receptor expression in unstimulated cells, 3) after bacterial exposure without ICS and 4) bacterial exposure with concurrent ICS. The MFI values are representative of the mean number of receptors per cell.
(TIF)

S2 Fig Heat map representation of baseline differences in receptor expression between subject groups. Heat map representation of baseline receptor expression. As analysis of log-transformed data generates (deceptively) small numerical differences representing significant changes, rainbow heat map representation is provided to clearly represent similarity or differences in receptor expression at baseline for the subject groups (COPD-ES, COPD-S, Sm, NoS). Only receptors found to be statistically significantly different between groups by ANOVA analysis had a heat map generated, with the ANOVA graph representation from the main manuscript, Fig 1A, presented to the right of each heatmap.
(TIF)

S3 Fig NTHI and SP-mediated reductions of bacterial recognition receptor expression on MDMs from subjects with COPD. Log transformed receptor expression reported as mean fluorescence intensity (MFI) is reported in appropriate quadrant for the subject group, at baseline and after NTHI and SP exposure. Statistically significant reductions in receptor expression after bacterial exposure, determined by paired T-test, are denoted by the double-sided arrow, with corresponding p-value. Rainbow heatmap legend (representing relative MFI by color) is to the right of each heatmap. Heatmap representation supports data that is presented in the main manuscript, Fig 2 and Table 2.
(TIF)

S1 File Supporting information. (DOCX)
