# Supplementary figures and images for: Bacterial regulation of macrophage bacterial recognition receptors in COPD are differentially modified by budesonide and fluticasone propionate
Source: PLoS One. 2019 Jan 24;14(1):e0207675. doi: 10.1371/journal.pone.0207675 (PMC6345465; doi:10.1371/journal.pone.0207675)

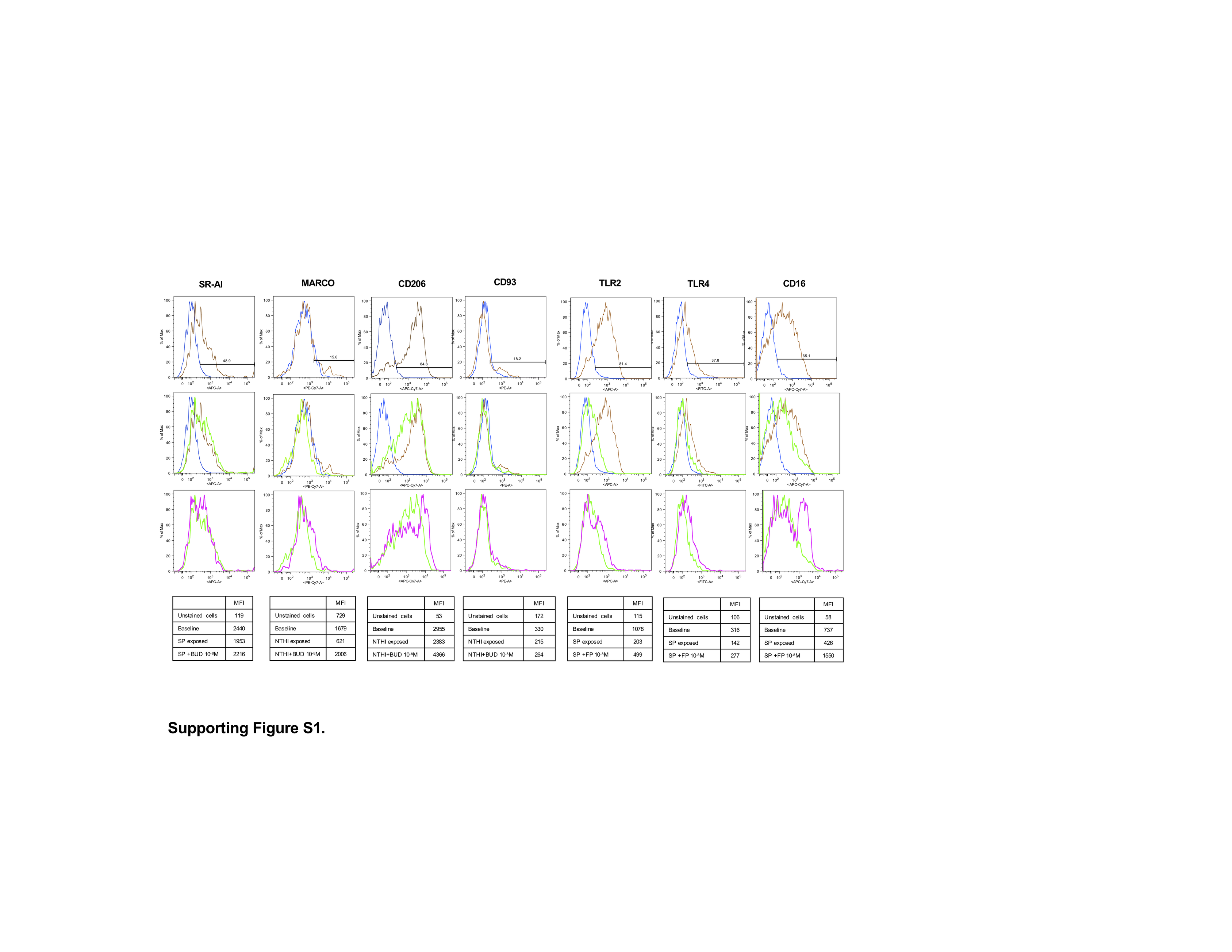

Supplement: S1 Fig — Histograms represent surface expression receptors gated on live COPD MDMs at baseline (brown histogram), after exposure to bacteria (green histogram) and ICS effects on receptor expression during exposure to bacteria (pink histogram), presented as mean fluorescence intensity (MFI). This is as described in the main text in Fig 2 (BUD) and Fig 3 (FP). X-axis is shown as the MFI, representative of the number of receptors per cell. Y axis represents the number (as percent) of the total number (Max). The top row demonstrates baseline MDM receptor expression (brown histogram) in MDMs not exposed to bacteria or ICS, as compared to unstained and unstimulated MDMs (cellular autofluorescence, blue histogram, biologic comparator). The black horizontal line and the number above reflect the gate used to determine the percent of cells with receptor expression detectable above cellular autofluorescence. The second row shows the effect of bacterial exposure on MDM receptor expression (green histogram) in relation to baseline expression (brown histogram) and autofluorescence (blue histogram) in unstained MDMs. SR-AI, TLR2, TLR4, CD16 represent the effect of SP on receptor expression; MARCO, CD206, CD93 represent effects of NTHI. The third row demonstrates receptor expression on MDMs pretreated with ICS and exposed to bacteria (pink histogram) in relation to receptor expression on MDMs not treated with ICS before bacterial exposure (green histogram). Effect of BUD is shown for SR-AI, MARCO, CD206 and CD93 (first 4 histograms, from left to right). Effect of FP is shown for TLR2, TLR4 and CD16 (last 3 histograms, from left to right). The tables in the bottom row provide the MFI for (from top to bottom) 1) unstained, unstimulated cells, 2) baseline receptor expression in unstimulated cells, 3) after bacterial exposure without ICS and 4) bacterial exposure with concurrent ICS. The MFI values are representative of the mean number of receptors per cell. (TIF) [file pone.0207675.s001.tif]

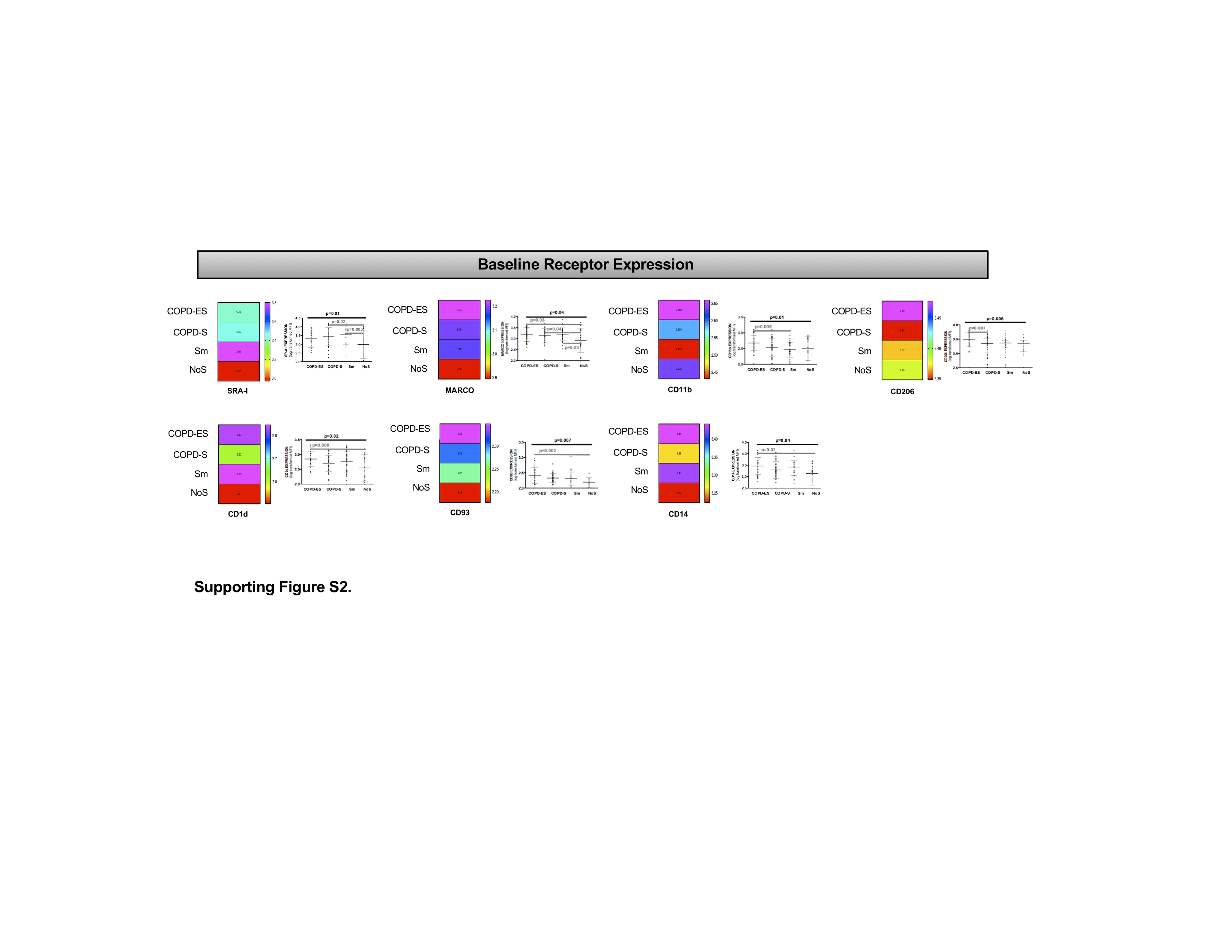

Supplement: S2 Fig — Heat map representation of baseline receptor expression. As analysis of log-transformed data generates (deceptively) small numerical differences representing significant changes, rainbow heat map representation is provided to clearly represent similarity or differences in receptor expression at baseline for the subject groups (COPD-ES, COPD-S, Sm, NoS). Only receptors found to be statistically significantly different between groups by ANOVA analysis had a heat map generated, with the ANOVA graph representation from the main manuscript, Fig 1A, presented to the right of each heatmap. (TIF) [file pone.0207675.s002.tif]

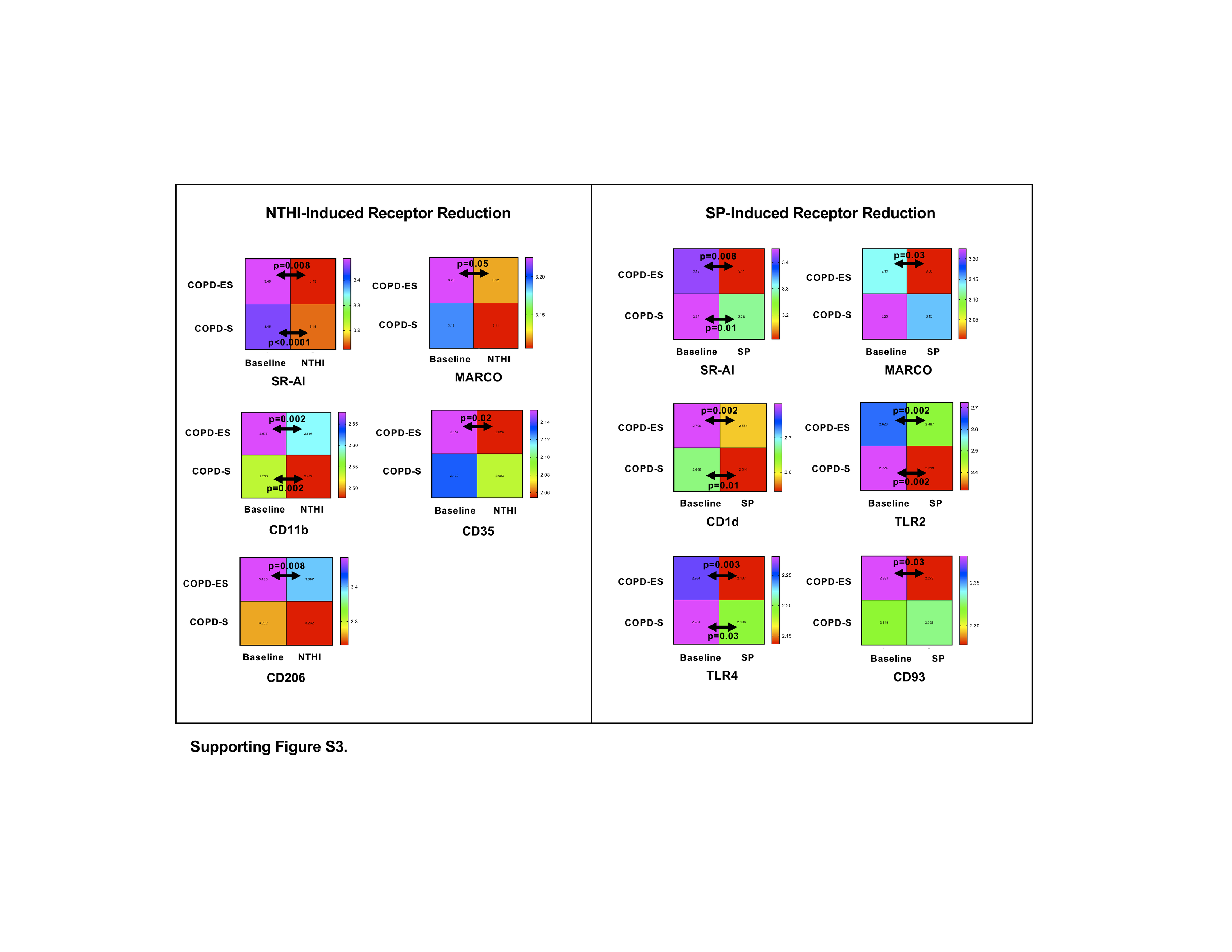

Supplement: S3 Fig — Log transformed receptor expression reported as mean fluorescence intensity (MFI) is reported in appropriate quadrant for the subject group, at baseline and after NTHI and SP exposure. Statistically significant reductions in receptor expression after bacterial exposure, determined by paired T-test, are denoted by the double-sided arrow, with corresponding p-value. Rainbow heatmap legend (representing relative MFI by color) is to the right of each heatmap. Heatmap representation supports data that is presented in the main manuscript, Fig 2 and Table 2. (TIF) [file pone.0207675.s003.tif]
