# Supplementary material for: Bacterial regulation of macrophage bacterial recognition receptors in COPD are differentially modified by budesonide and fluticasone propionate
Source: PLoS One. 2019 Jan 24;14(1):e0207675. doi: 10.1371/journal.pone.0207675 (PMC6345465; doi:10.1371/journal.pone.0207675)
Supplement: S1 File — (DOCX) [file pone.0207675.s004.docx]

**Bacterial regulation of macrophage bacterial recognition receptors in COPD are differentially modified by budesonide and fluticasone propionate**

**Online Supplement Methods**

**Recruitment of Subjects**

The investigator-initiated study (AstraZeneca study D589BN00039, Veterans Affairs Western New York Healthcare System at Buffalo (VA WNY) (study 466402) included subjects with moderate to severe stable COPD, which was defined as > 10 pack year history of smoking, who were either current (COPD-S) or ex-smokers (COPD-ES), had evidence of fixed airflow obstruction with a FEV1/FVC < LLN (lower limit of normal), and FEV1<65%. Non-smoker healthy controls (NoS) were defined as <5 pack-year smoking history (only 1 of the 10 subjects had any tobacco exposure, with a 3 pack-year total giving a mean pack year of 0.3 years and median of zero), not currently smoking and demonstrating normal spirometry. Smokers without COPD (Sm) were defined as having normal spirometry, currently smoking, with a >10 pack-year history of cigarette use. Although attempts were made to age match the Sm to the COPD groups, many of the subjects recruited for the age-matched Sm group ultimately entered the study in the COPD-S group, identified on initial screening spirometry. Similar age-matching difficulties occurred in the non-smoker group (NoS), with the most common reason for exclusion of candidates of similarly advanced age were anemia and restrictive lung disease on pulmonary function testing due to heart failure. As discussed in the main manuscript, the difficulties of finding older healthy smokers have been seen in other studies similar to ours (manuscript references 25, 29, 34). Berenson et al (manuscript references 25, 34) also looked for effect of age on outcomes by regression analysis and found no effect, consistent with our results.

Exclusion criterion included requirement of greater than 5 liters per minute oxygen supplementation, use of immunosuppressant medications, concomitant diagnoses of anemia, blood disorders or lung disease other than COPD. Patients with COPD were not enrolled within 90 days of an exacerbation, were not taking oral steroids and were felt to have a stable health status at the time of study enrollment. One hundred milliliters of blood was obtained from each qualified subject on 2 separate occasions, separated by at least 30 days (but no more than 90 days), to allow for recovery of red blood cell numbers between blood draws, as approved by the VA WNY Institutional Review Board. Target enrollment was 40 subjects with COPD (20 current smokers and 20 ex-smokers), 20 smokers and 10 non-smoker healthy controls. One subject with COPD who was smoking at the time of study enrollment, quit smoking for > 30 days before her first blood draw, and crossed over into the ex-smoker group, leading to the uneven enrollment of 21 COPD-ES and 19 COPD-S.

**Purification of blood monocytes**

Whole blood was withdrawn from each participant into 10 heparinized tubes. An equal volume of HBSS was added to the whole blood, layered with lymphocyte separation media (Mediatech, Herndon, VA USA) and centrifuged. The resulting leukocyte layer was removed. Percent viability was determined by trypan blue exclusion. Leukocytes underwent further negative bead depletion (Dynabeads, Invitrogen/Life Technologies, Grand Island, NY, USA) to yield a pure monocyte population (>95%) using mouse anti-human antibodies against CD2 (mouse IgG1,κ, clone RPA-2.10), CD3 (mouse IgG2a,κ, clone HIT3a), CD19 (mouse IgG1,κ, clone HIB19), CD56 (mouse IgG1,κ, clone B159), CD66 (mouse IgG2a, κ, clone B1.1/CD66) and CD235a (mouse IgG2b, κ, clone GA-R2) (BD Biosciences, San Jose, CA USA). Purity of cells was confirmed using flow cytometric analysis for residual contaminating cell populations, H&E staining and direct visualization. Final yield of purified monocyte concentrations was determined after negative depletion by trypan blue exclusion.

**Generation of monocyte-derived macrophages**

Isolated monocytes were cultured in suspension culture in low adherence flasks (Corning Tewksbury, MA USA) in RPMI without phenol red, supplemented with 10% FBS, 1% pen-strep, 1% L-glutamine, 2.5 mM Hepes and 20 ng/mL GM-CSF shown previously to effectively generate monocyte-derived macrophages [1, 2], lacking the characteristic markers of dendritic cell populations [1]. All media components were purchased to be free of contaminating LPS. Cells were incubated at 37^o^C with 5% C0_2_. Additional media was added at day 4 and changed entirely on day 7. Mature monocyte-derived macrophages (MDMs) were harvested for use at day 11.

**Monocyte-derived macrophage incubation with bacteria**

MDMs were washed and re-suspended in serum free, phenol red free HBSS without calcium or magnesium for 12 hours to reduce any stimulatory effect from the serum. They were pre-treated with the glucocorticoids budesonide (BUD) or fluticasone propionate (FP) (both in 0.1% DMSO), with a concentration range of 1 nM to 10 µM, or 0.1% DMSO alone for 24 hours. The concentrations of dissolved glucocorticoids were chosen based on clinically relevant airway/lung tissue concentrations of 1-100 nM after inhalation[3-5], and a range above to assess the concentration-response relationship and drug toxicity. It was pre-determined that the studied concentrations would be narrowed to 3 on the most linear part of the concentration-response curve, based on preliminary experiments. The 10 µM concentration was not continued due to significant cellular toxicity observed with both BUD and FP. The 1 nM concentration was not continued due plateau effect on cytokine release below 10 nM. The most consistent results were observed at 10 nM, which is considered to be the most clinically relevant concentration in airway and lung tissue during the several-hour period after inhalation[3-5], and all subsequent analyses were performed using this concentration.

Clinical isolates of live non-typeable *Haemophilus influenzae* (NTHI) (strain 11P6H) or *Streptococcus pneumoniae* (SP) (strain 67PP3) were then added to the media containing BUD, FP or diluent control at log-phase with a final projected MOI of 200:1 bacteria: cells after a dose titration to achieve maximal bacterial uptake with minimal cell death, with the optimal ratio resulted identical to previous work[2, 6, 7]. SP co-cultures additionally received 5% antibody depleted serum necessary for phagocytosis of SP. The bacteria and cells were incubated at 37^o^C on a rotational shaker for 24 hours. At 8 hours after bacterial inoculation, pen/strep at 1% volume/volume concentration was added, and the culture continued out to 24 hours. Cell surface receptor expression and supernatant cytokine analysis were evaluated at the end of the 24-hour co-culture incubation period.

**Flow cytometric analysis of cell surface receptors**

Multi-color flow cytometry for the cell surface receptors with monoclonal antibodies directed against CD14 (mouse IgG1, κ, clone 61D3), CD35 (mouse IgG1, clone E11), CD93 (mouse IgM, clone R3), TLR4 (mouse IgG2a, κ, clone HTA125) (ebiosciences San Diego, CA USA); CD16 (mouse IgG1, κ, clone 3G8), TLR2 (mouse IgG2a, κ, clone TL2.1), CD206 (mouse IgG1, κ, clone 15-2), CD11b (mouse IgG1, κ, clone ICRF44), CD1d (mouse IgG2b, κ, clone 51.1)(BioLegend San Diego, CA USA); SRA-I (mouse IgG2b, clone 351615) (R&D Systems Minneapolis, MN USA); MARCO (mouse IgG3, clone PLK-1) (Hycult Biotech, Plymouth Meeting, PA) was performed on the MDMs. Effective FRET transfer was confirmed and optimized for all tandem dyes to prevent spillover into the adjacent channels (i.e. APCCy7 into APC, PECy7 into PE). Flow cytometer calibration and spillover was assessed for each experiment and each tandem conjugate, using antibody capture beads (BD Pharmingen). Biologic comparators (unstained, unstimulated control MDMs from each subject) were used instead of isotype controls to distinguish positive from negative events, given the limitations of isotype controls in multicolor flow cytometry with tandem dyes wherein variability in the fluorophore: antibody ratio, degree of isotype antibody aggregation, concentration and FRET transfer rates that differ from the target antibody make isotypes an unreliable control[8, 9]. The mean fluorescence intensity (MFI) was taken to represent relative receptor density in the final analysis.

PE and PE-Cy7 antibody fluorophore labels were found to overlap within the MDM cellular autofluorescence at voltages required for effective FRET transfer. The intensity of the autoflourescence in the far-red spectrum of PE-Cy7 required a conservative gating strategy, to ensure that cellular autofluorescence could be accurately distinguished from true positive results. We accepted a lower rate of positive receptor determination, for greater accuracy, accepting < 20% histogram overlap between positively stained and unstained cells, and setting histogram gates based on dot plot overlays to ensure absence of false positives. Data analysis was performed using Flowjo software for Mac (Treestar, Ashland, OR USA).

**Statistical Analysis**

Statistical analyses were completed using Prism 7 (GraphPad San Diego, CA USA). As the data sets, except demographic data, were non-normally distributed with significant skewness and kurtosis, the data was therefore log transformed prior to analysis. Subsequent analysis was performed by parametric analyses, using repeated measures one-way ANOVA , T-test (paired or unpaired) and linear regression with a two-tailed p value of <0.05 regarded as statistically significant. To address issues of false-discovery rates, Holm-Sidak correction for multiple comparisons was performed for ANOVA analyses and adjusted p-values are reported; Benjamini-Hochberg correction was used when multiple independent testing (age-receptor linear regression, receptor response to bacteria) was performed. Heat map representation of NTHI and SP reductions in baseline receptor expression is presented to visually support the stastically significant differences. Linear trend analysis for the responses to 10 nM, 100 nM and 1 μM drug concentrations from the one-way ANOVA was done to determine linear part of concentration-response relationship. Given the relatively small sample size and potential for type II error (i.e., failure to reject a false null hypothesis), emphasis was also placed on Cohen’s-d effect sizes[10]. Cohen’s-d testing is used as a counter-point to significance tests, which defines only a greater than 0 difference between groups that reaches statistical significance. The effect size allows detection of a significant effect of the intervention with small sample sizes, and defines the magnitude of the result as it occurs in the population (how big or small a significant difference is).

**References**

1. Winkler AR, Nocka KH, Sulahian TH, Kobzik L, Williams CM. In vitro modeling of human alveolar macrophage smoke exposure: enhanced inflammation and impaired function. Exp Lung Res. 2008;34(9):599-629. Epub 2008/11/14. doi: 905449346 [pii]

10.1080/01902140802366261. PubMed PMID: 19005923.

2. Provost KA, Smith M, Arold SP, Hava DL, Sethi S. Calcium restores the macrophage response to nontypeable haemophilus influenzae in chronic obstructive pulmonary disease. Am J Respir Cell Mol Biol. 2015;52(6):728-37. doi: 10.1165/rcmb.2014-0172OC. PubMed PMID: 25338285.

3. Esmailpour N, Hogger P, Rabe K, Heitmann U, Nakashima M, Rohdewald P. Distribution of inhaled fluticasone propionate between human lung tissue and serum in vivo. Eur Respir J. 1997;10:1496-9.

4. Van den Bosch JM, Westermann CJ, Aumann J, Edsbacker S, Tonnesson M, Selroos O. Relationship between lung tissue and blood plasma concentrations of inhaled budesonide. Biopharmaceutics & drug disposition. 1993;14(5):455-9. Epub 1993/07/01. PubMed PMID: 8218963.

5. Maassen van den Brink KI, Boorsma M, Staal-van den Brekel AJ, Edsbacker S, Wouters EF, Thorsson L. Evidence of the in vivo esterification of budesonide in human airways. British Journal of Clinical Pharmacology. 2008;66(1):27-35.

6. Berenson CS, Garlipp MA, Grove LJ, Maloney J, Sethi S. Impaired Phagocytosis of Nontypeable Haemophilus influenzae by Human Alveolar Macrophages in Chronic Obstructive Pulmonary Disease. The Journal of Infectious Diseases. 2006;194:1375-84.

7. Berenson CS, Wrona CT, Grove LJ, Maloney J, Garlipp MA, Wallace PK, et al. Impaired Alveolar Macrophage Response to Haemophilus Antigens in Chronic Obstructive Lung Disease. Am J Respir Crit Care Med. 2006;174:31-40.

8. Maecker HT, Trotter J. Flow cytometry controls, instrument setup, and the determination of positivity. Cytometry Part A : the journal of the International Society for Analytical Cytology. 2006;69(9):1037-42. Epub 2006/08/05. doi: 10.1002/cyto.a.20333. PubMed PMID: 16888771.

9. Keeney M, Gratama JW, Chin-Yee IH, Sutherland DR. Isotype controls in the analysis of lymphocytes and CD34+ stem and progenitor cells by flow cytometry--time to let go! Cytometry. 1998;34(6):280-3. Epub 1999/01/08. PubMed PMID: 9879645.

10. Cohen J. Statistical power for the behavioral sciences. 2nd edition ed. Hillsdale, NJ: Lawrence Erlbaum Associates; 1988.
